# Changes in Health and Their Relationship to Paid Work Across Older Cohorts in England

**DOI:** 10.1093/geroni/igaf122.1344

**Published:** 2025-12-31

**Authors:** Karen Glaser

**Affiliations:** King’s College London, London, England, United Kingdom

## Abstract

This paper investigates the role of mental distress and other forms of ill-health/disability as drivers of increases in economic inactivity among older workers. Economic inactivity among 50–64-year-olds has risen since the pandemic (2019-2022) and has yet to recover to pre-pandemic levels (ONS 2023). This rise is not seen in any other high-income country and reverses the rise of UK employment in this age group since the mid-1990s. It has been suggested that this rise in economic activity is due to increased levels of poor health; however, such findings are based on responses to questions about the ‘main reasons’ for economic inactivity rather than health-related questions. Thus, we know little about whether changes in mental distress and other forms of ill-health (if any) are driving economic inactivity levels among older adults and for which groups (e.g., low-income households). Employing data from the English Longitudinal Study of Ageing, we investigate changes across cohorts in: (1) mental distress and other forms ill-health/disability (drawing on work and health-related measures) and their relationship with paid work; (2) the nature of long-term sickness (and individual and family circumstances), and implications for paid work; and (3) how people respond to ill-health/disability through multiple types of work transitions (e.g., exiting employment, reducing work hours, changing jobs) and depending on working conditions.

